# Ultrasonic Processing for Structure Refinement: An Overview of Mechanisms and Application of the Interdependence Theory

**DOI:** 10.3390/ma12193187

**Published:** 2019-09-28

**Authors:** Nagasivamuni Balasubramani, David StJohn, Matthew Dargusch, Gui Wang

**Affiliations:** 1 Centre for Advanced Material Processing and Manufacturing (AMPAM), School of Mechanical and Mining Engineering, The University of Queensland, St Lucia 4072, Australia; n.balasubramani@uq.edu.au (N.B.); d.stjohn@uq.edu.au (D.S.); m.dargusch@uq.edu.au (M.D.); 2 DMTC Limited, The University of Queensland, St Lucia 4072, Australia

**Keywords:** ultrasonic treatment, grain refinement, interdependence model, aluminium alloys, magnesium alloys, zinc

## Abstract

Research on ultrasonic treatment (UST) of aluminium, magnesium and zinc undertaken by the authors and their collaborators was stimulated by renewed interest internationally in this technology and the establishment of the ExoMet program of which The University of Queensland (UQ) was a partner. The direction for our research was driven by a desire to understand the UST parameters that need to be controlled to achieve a fine equiaxed grain structure throughout a casting. Previous work highlighted that increasing the growth restriction factor *Q* can lead to significant refinement when UST is applied. We extended this approach to using the Interdependence model as a framework for identifying some of the factors (e.g., solute and temperature gradient) that could be optimised in order to achieve the best refinement from UST for a range of alloy compositions. This work confirmed established knowledge on the benefits of both liquid-only treatment and the additional refinement when UST is applied during the nucleation stage of solidification. The importance of acoustic streaming, treatment time and settling of grains were revealed as critical factors in achieving a fully equiaxed structure. The Interdependence model also explained the limit to refinement obtained when nanoparticle composites are treated. This overview presents the key results and mechanisms arising from our research and considers directions for future research.

## 1. Introduction 

Our interest in the potential of UST to refine the grain structure of metals began in 2008 when it was realised that alloy composition can have a significant effect on grain size even under the application of UST [1]. It was found that grain size is linearly related to the inverse of the growth restriction factor *Q* in line with observations for alloys cast by traditional casting processes [1,2,3]. The other incentive was the increase in interest shown by industry and academia to use UST to potentially refine the grain size to below that achieved by the addition of inoculant particles, or to eliminate the use of inoculant containing master alloys such as Mg-Zr for Al-free magnesium alloys which are expensive and wasteful [4,5,6]. Further stimulus came from partnering with the European project ExoMet in 2012 that focused on the development of new liquid metal processing techniques using external fields (mainly UST) on Mg and Al based light alloys and nanocomposites. The initial focus of our research was to understand the role of alloy composition in relation to the effectiveness of UST as a grain refinement method. In the process we gained knowledge about the mechanisms and parameters that need to be controlled to ensure UST provides consistent grain refinement throughout a casting. Thus, after a decade of research on a number of alloy systems and compositions, it is a good time to review this work as a whole with some new experimental results in the expectation that further insights will be revealed.

Controlling the as-cast grain size of castings is one of the important steps for achieving the desired quality and mechanical properties of a cast product [7,8]. Fine, equiaxed and non-dendritic grain structures in castings provide uniform mechanical properties, improved distribution of secondary phases and enhanced feeding to eliminate shrinkage porosity [8,9,10,11]. In the case of ingot or continuous casting, a refined uniform grain structure can significantly improve the downstream processing ability and productivity such as forging, rolling and extrusion operations by reducing the risk of hot tearing and macro-segregation [9,11]. For instance, producing a fine grain microstructure in Mg alloys throughout the thickness of the sheet without composition variation is critical for manufacturing high quality sheet by twin-roll casting [12]. 

Generally, grain refinement is induced by the addition of a master alloy containing heterogeneous nucleating particles. For example, the addition of Ti and Zr to specific Al and Mg alloys produces excellent refinement in grain size of cast products [8,13,14]. Although the addition of grain refining master alloy is the most common foundry practice, there are several disadvantages including (i) low efficiency where only a few percent or even less of the added particles are active in grain nucleation [15,16]; (ii) refiners have only been identified for certain alloy systems [8,13,14]; (iii) difficulty in uniformly distributing the refiner particles into the melt without the formation of agglomerates; (iv) interaction of nucleant particles with impurity elements leading to a poisoning effect [17,18] or grain coarsening by the addition of the alloying element itself (e.g., Si poisoning in Al alloys) [19]; (v) fading due to the loss of the refinement ability by particle settling; and (v) the high cost of master alloys [5,6]. 

Liquid and semi-solid melt processing with power ultrasound has received considerable attention and has been successfully demonstrated for the refinement of as-cast grain structure in light alloys without the need for grain refiners, therefore eliminating the limitations associated with inoculation processes for Al and Mg based alloys [20,21,22]. The benefits of ultrasonic treatment (UST) include the production of non-dendritic structures, degasification and the refinement of primary phases (Al_3_Zr, Al_3_Ti, Al-Fe-Si and primary Si) [23,24,25]. This technique has attracted commercial interest due to being environmentally friendly, cost effective and possesses several technical advantages over conventional methods in molten metal processing [23,26,27].

Earlier work by Eskin [22,23] and Abramov [28] showed the potential of UST to refine the grain structure in various alloys and its possible mechanisms. The occurrence of cavitation (nucleation and implosion of bubbles during alternate pressure cycles of ultrasound) and acoustic streaming were identified as two key phenomena that change the dynamics of solidification. Significant effort has been made by Eskin and co-workers to understand the role of cavitation and acoustic steaming using advanced X-ray synchrotron solidification techniques and by modelling the dynamics of cavitation bubble formation [29,30,31,32,33,34]. A recent review by Eskin et al. [34] provided a broad summary of the effects of cavitation and acoustic streaming (mainly on in-situ observations and ex-situ solidification studies) on heterogeneous nucleation, fragmentation of primary crystals and intermetallic phases and de-agglomeration mechanisms. Alternative to the in-situ radiography techniques, observation of organic transparent analogues (e.g., succinonitrile (SCN)-1 wt.% camphor alloy) using a high-speed camera during UST also reveals the streaming flow pattern and the interaction of cavitation bubbles with dendrites and the mechanisms of fragmentation [35]. These in-situ solidification studies have increased our current understanding by elucidating the role of cavitation bubbles and explained the importance of these mechanisms for the upscaling of UST to large melt volumes in industrial applications. Considerable research has also highlighted the importance of cavitation and acoustic streaming on the activation of potent particles, nucleation of grains, altering convection patterns and reducing the temperature gradients during solidification [23,26,27,28,36,37,38,39,40]. While recent publications on advanced (in-situ) solidification studies have provided more detailed evidence on bubble dynamics during melt solidification [31,32,33,41,42], our research has been focused on other factors such as solute content, type of solute, constitutional supercooling, role of potent and impotent (oxide) particles, UST duration, origin and transport of grains, temperature range over which UST is applied, sonotrode preheating and other casting variables that could affect grain formation [6,37,38,43,44,45,46,47]. Using the Interdependence Theory of nucleation and grain refinement [15,48], we have revealed explanations for the role of solute [37,38,45,49], casting conditions, and micro [6,46] and nanoparticles [50] in assisting refinement by the application of UST. 

This paper begins with a brief description of aspects of the experimental design such as the choice of metals, alloys and master alloys that were investigated, experimental techniques involved in the casting process, control of UST variables, modelling and simulation of acoustic streaming, and analytical methods. The results of these experiments are presented for two situations: UST applied above the liquidus temperature and UST applied from above to below the liquidus to include the nucleation stage of solidification. This is followed by an analysis of the origin and transport of grains, role of alloy elements and then an analysis of the grain nucleation mechanisms using the Interdependence model.

## 2. Experimental Design

### 2.1. Metals and Alloys Investigated

To investigate the effects of alloy composition, temperature range and duration of the application of UST, and the refinement mechanisms operating during solidification, different alloy systems were chosen from pure metals (Al, Mg, Zn), alloys (Al-Cu, Al-Si, Al-Mg) and alloys with the addition of master alloys: Al and its alloys with AlTiB master alloy, Mg and its alloys with Mg-Zr master alloy. The alloys and pure metal ingots were cast from commercial purity Al (99.7%), high purity Zn (99.995%), commercial purity Mg (99.91%), copper (99.9%) and silicon (99.4%). Al alloys with varying concentration of Si [45], Cu [37,51,52] and Mg [49] were cast with and without UST. Master alloys of Al3Ti1B and Mg-25Zr were introduced into the melt to understand the effect of nucleant particles [6,46]. Approximately 135 to 210 cc of pure metals and alloys were melted and solidified in a clay-graphite crucible in most of the experiments presented in [6,43,44,47,49,52]. The melt temperature was chosen to ensure there was enough superheat to overcome the chill effect induced by the unpreheated sonotrode. This ensured that the sonotrode was heated sufficiently to establish acoustic streaming in the melt before solidification began.

### 2.2. Application of UST

**Figure 1:**
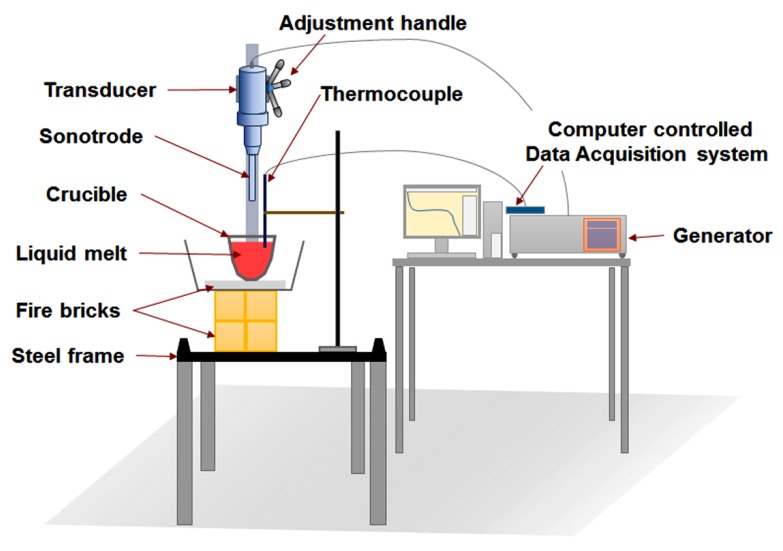
Ultrasonic treatment (UST) platform (steel frame) shows the arrangement of a crucible containing liquid melt placed over fire bricks. The adjustable handle is used to lower the sonotrode into the melt when the required temperature is recorded by the data acquisition system (K-type thermocouple).

The experimental set-up is illustrated in Figure 1. An ultrasonic system (Sonics VCX1500, 20 kHz and 1.5 kW, Sonics & Materials, Inc., Newtown, CT, USA) made of a piezoelectric transducer, power generator, air cooling unit and an adjustment handle for lowering the sonotrode into the melt. A fixed power of 40% of the total power was used in all the UST experiments. The sonotrode was made of titanium alloy or molybdenum alloy designed for half a wavelength (λ/2 = 125 mm) having a diameter of 19 mm. After melting, the crucible containing the liquid metal from the furnace, was transferred to the platform and cooled in air as shown in Figure 1 [44]. The platform also has provision to measure the cooling curves using K-type thermocouples. The sonotrode (sometimes at room temperature or preheated to 285 °C) was turned on in air and then inserted into the melt at the required temperature, which then solidified in air for a specified duration. Table 1 shows the details of the pure metals and alloys investigated for refinement of grains and primary intermetallic phases classified according to the alloy type with a short summary of the outcomes of the work. Using the fixed value of ultrasonic power, grain refinement was studied with respect to temperature range and time duration of UST. For the casting conditions and alloys investigated in the present work, the UST duration was varied from 30 s to 4 min in Mg and Al castings and 2 to 9 min in Zn castings. To account for the effect of volume, three additional large volume castings (~322, 530, 946 cc) of Al-2Cu alloys were solidified under UST in order to understand the effect of casting volume and height (H < λ/2, H = λ/2 and H > λ/2) on the refinement of grain structure.

### 2.3. Macro and Micro Structure Examination

Solidified samples with and without UST were sectioned vertically to analyse the macrostructure and small samples of (15 mm × 10 mm) were taken to analyse the microstructural refinement. After polishing the samples using the standard metallographic preparation methods and etching, microstructures were examined in a polarised light microscope (Leica Polyvar). The grain size measurements were calculated using the linear intercept method by image analysis software [6,43]. The specific procedures of the composition analysis, sampling regions, etchants and etching techniques used for individual alloy systems can be found in the respective references listed in Table 1.

### 2.4. Modelling and Validation of Acoustic Streaming 

Theoretically, acoustic streaming can be considered as a time averaged transfer of momentum flux per unit area that creates a non-uniform accelerating flow which converts the wave energy into fluid motion [54]. A jet of streaming flow during solidification could significantly affect solute diffusion, convection and transport phenomena at the solid-liquid interface, which in turn is associated with dendritic fragmentation, grain growth, instabilities and the morphological features of grains [55,56]. The flow pattern induced by the acoustic stream can be tracked visibly using transparent analogues such as water, glycerine and ethanol [57]. In the case of liquid melts, it is difficult to directly observe the flow pattern induced by acoustic streaming, however, with reference to the transparent analogues, numerical solvers can be used to predict acoustic streaming and its effect on temperature gradient, flow velocity and acoustic pressure gradients [37,38,58,59,60,61,62].

Acoustic streaming induced fluid flow during UST was modelled by Wang et al. [38] using momentum equations in the ProCAST simulator that drives the net acoustic forces generated by the radiating surface propagating into the liquid using the Reynolds turbulent wave equation. The model aimed to identify the impact of forced flow in the melt affecting the temperature distribution of Al-2Cu alloy solidified in clay graphite-crucible (210 cc volume). Specific dimensions and heat transfer coefficients can be found in the ref [38]. Acoustic streaming was calculated using Navier–Stokes model at high Reynolds number as

(1) $$\rho (\vec{v}\cdot \nabla \vec{v})=-\nabla \bar{p}+\mu \nabla^{2}\vec{v}+{\vec{F}}_{N}$$

where, ρ is density, $\bar{p}$ is the mean pressure and μ is the kinematic viscosity. It is assumed that the powerful streaming was created beneath the sonotrode tip and forced into the liquid at a velocity v, attenuating at a constant rate (P) with increasing distance from the tip of the sonotrode. The net force acting on the fluid exerts a momentum force along a particular direction (x) and is given by

(2) $${\vec{F}}_{N}=\frac{P}{c}(1-e^{-\beta x})$$

Here β is the attenuation coefficient. The resultant kinematic momentum (K) is given by

(3) $$K=\rho \cdot {\vec{F}}_{N}$$

**Figure 2:**
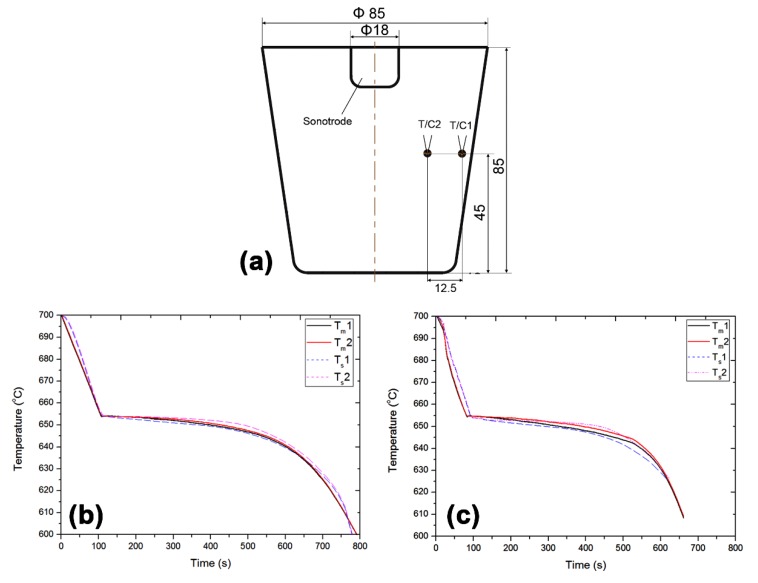
(**a**) Schematic of the casting setup with two thermocouples for the validation of the model and the corresponding cooling curves in (**b**) as-cast condition without UST and (**c**) after UST [38].

A momentum source of approximately 2.3 × 10^-6^ m^3^ located beneath the sonotrode induces a net force of 0.024 N along the principal direction of the acoustic streaming (vertically downwards). The convective motion is then modelled using Navier–Stokes equations coupled with energy equations [38]. Figure 2a shows the casting setup with two thermocouples placed at the mould wall (T/C1) and the off-centre position (T/C2) to validate the current model. The cooling curves in Figure 2b,c show a good correlation between the measured (T_m_) and simulated (T_s_) temperature measurements in the as-cast condition and after the application of UST. This also validates the current assumption regarding the attenuation rate (P/c) of the momentum source (K), which is used to simulate the temperature gradients in the casting during solidification. This modelling approach does not include the incorporation of the effect of cavitation, simulation of grain size as a result of fragmentation and grain transport due to acoustic streaming [37,38].

## 3. Simulation of Acoustic Streaming

**Figure 3:**
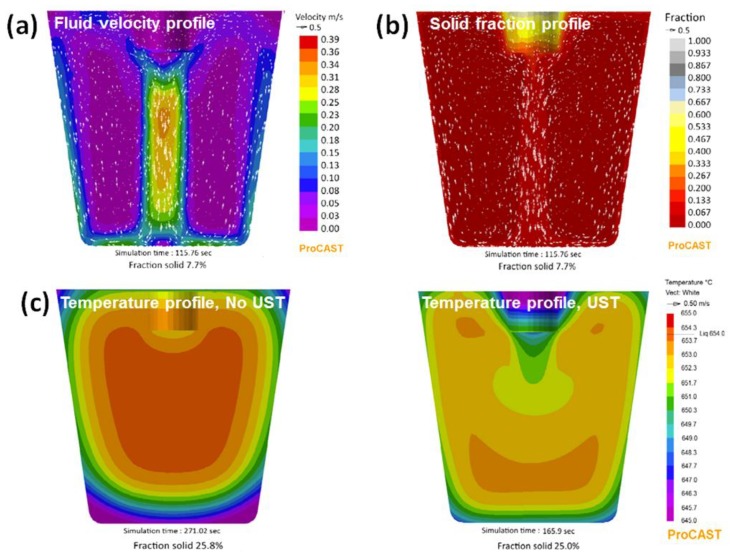
Simulations of (**a**) Fluid velocity and (**b**) solid fraction during UST, and (**c**) temperature gradient before and after UST in Al–2Cu alloy [38].

Figure 3 shows the simulated velocity, solid fraction and temperature profile under a momentum induced acoustic streaming model. The simulation results showed that the fluid velocity varies from 0.38 m/s at the start of solidification producing a nearly flat temperature gradient and decreases to 0.32 m/s with the increase in solid fraction during solidification. The fluid velocity profile in Figure 3a shows a maximum velocity directly beneath the sonotrode and the recirculation pattern shows a high fluid velocity at the mould wall regions. Figure 3b shows the solid fraction profile corresponding to the velocity map shown in Figure 3a taken at the same solid fraction of 7.7%. It is revealed that a higher solid fraction contour is found at the centre region beneath the sonotrode, indicating that the first to form solid lies beneath the sonotode which gets pushed downstream by the action of acoustic streaming. Figure 3c shows the temperature distribution profile in as-cast and during UST conditions. Without UST, the mould walls of the crucible extract heat rapidly from the melt and the temperature distribution shows a steep gradient from the wall region to the centre of the crucible. After UST, the simulated profile shows almost a flat temperature with reduced gradient from the mould wall and the coldest zone being the cavitation zone where the grains are being produced and dispersed. By correlating the simulation results with the experimental grain size results, it was found that the high fluid velocity created by acoustic streaming tends to flatten the temperature gradient promoting the formation of an equiaxed grain structure [37,38].

**Figure 4:**
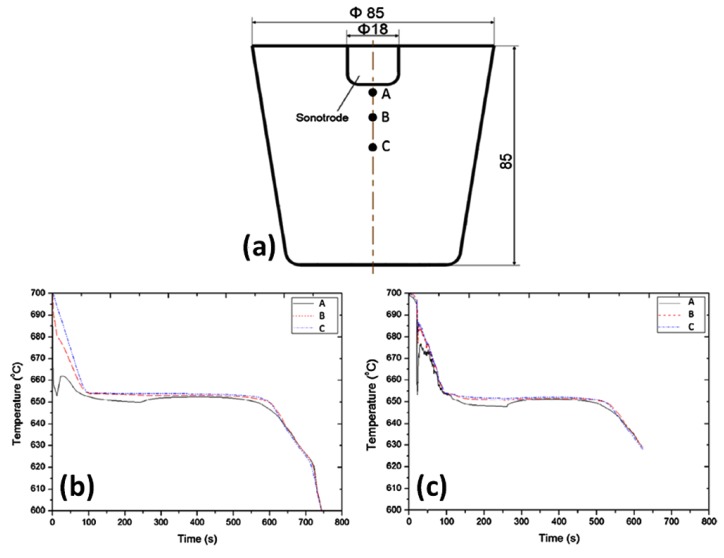
(**a**) Schematic of the casting setup shows the locations of points (A, B, C) and the simulated cooling curves of an Al-2Cu alloy when (**b**) the sonotrode is immersed into the melt but is not turned on during solidification and (**c**) the sonotrode is turned on before immersion into the melt and remains on during solidification as detailed in [38].

The flow fields affecting the temperature gradient can also be understood by cooling curve analysis during the solidification process. Figure 4a shows the simulated solidification profile at three points (A to C). The cooling curves from the corresponding points are shown in Figure 4b when the sonotrode is immersed into the melt but without turning on the sonotrode and Figure 4c where the sonotrode is turned on before immersion into the melt. After inserting the idle sonotrode into the melt the location at C and B is almost undisturbed while cooling curve A shows a significant drop in the temperature. Location A in Figure 4a represents the cavitation zone beneath the sonotrode where the observed temperature drop is 660 °C, which is only 5 °C above the liquidus temperature of the Al-2Cu alloy [52]. When the temperature drops below the liquidus the cavitation zone is referred to as undercooled zone, which is also confirmed by the simulation (Figure 3b,c). When UST is initiated, all the cooling profiles appear to be similar (Figure 4c). Cooling curve A (solid line) almost matches curve B after UST indicating that the bulk melt temperatures are the same allowing the powerful acoustic streaming to stabilise the temperature in A, B and C enabling the transport of the new grains formed beneath the sonotrode into the bulk melt (Figure 4c). 

This stabilised bulk temperature reduces the risk of remelting grains and retains the non-dendritic structure after solidification. 

## 4. UST Applied to the Liquid Melt

In liquid melt, UST can be applied in two ways, either isothermally treated at a specific melt temperature and then poured into the mould [2,63,64] or treating the liquid melt over a temperature range during continuous cooling and terminated before the onset of primary phase nucleation at the liquidus temperature [6,47]. The melt above the liquidus temperature (superheated condition) always contains insoluble impurities (mainly oxides) that are not actively involved in nucleation under the normal casting conditions [23]. The occurrence of cavitation in the melt is advantageous by wetting these un-wetted particles turning them into active nucleation sites for grain refinement [23,27]. 

### 4.1. Pure Metals and Alloys

**Figure 5:**
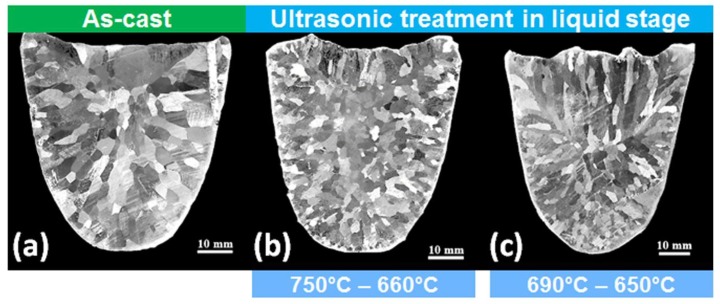
Macrostructure of commercial purity Mg in (**a**) without UST and after UST terminated at (**b**) 660 °C and (**c**) 650 °C [44].

UST of pure Al does not result in significant refinement, however, it has been reported that Al-11Cu and Al-4Cu alloys showed approximately 20% to 25% refinement in the grain size. With the intentional addition of Al_2_O_3_ particles it was found that UST refinement in the liquid condition is governed by cavitation enhanced activation of heterogeneous oxide particles [2]. Figure 5 shows the macrostructures of pure Mg before and after UST terminated at 10 °C above and at the melting temperature respectively. Approximately 25% to 28% refinement is observed compared to the as-cast structure, however, the degree of refinement is not significant (Figure 5b,c). It is interesting to note that the grain orientation is non-uniform after UST, while in the as-cast condition the grains are oriented opposite to the direction of heat extraction. A similar observation has been reported for the Al-2Cu alloy when UST is applied from 714 °C to 660 °C (liquidus temperature = 655 °C) resulting in an insignificant reduction in grain size. The observed refinement could be associated with oxides or native impurity particles [44]. 

### 4.2. With the Addition of Refining Master Alloys 

**Figure 6:**
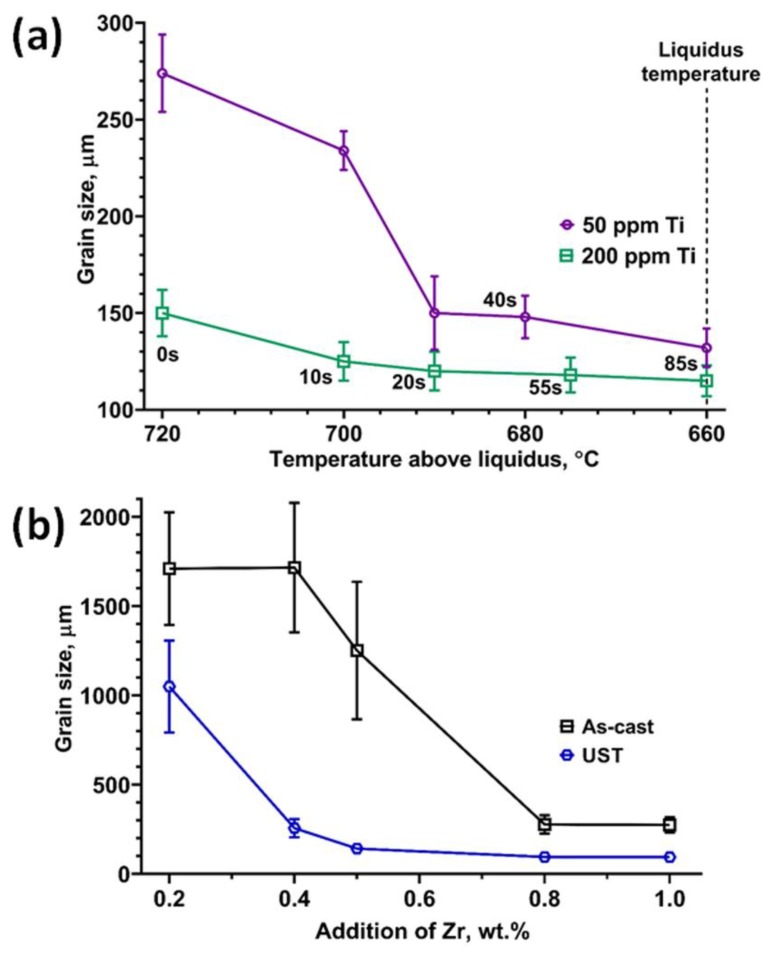
UST terminated above liquidus temperature at specific temperatures from 720 °C to 660 °C in (**a**) Al_3_Ti1B master alloy added at 50 and 200 ppm to pure Al and (**b**) Mg-25Zr master alloy added to commercial purity Mg. The liquidus temperature in Mg-Zr alloys varies from 651 °C to 653 °C in which UST is applied from 750 °C to 660 °C for 60 to 90 s.

Wang et al. [47] investigated the effect of adding Al_3_Ti1B master alloy to pure Al by terminating UST at specific temperatures above the liquidus temperature during continuous cooling (from 720 °C to 660 °C). Figure 6a shows the grain refinement obtained after the addition of 50 and 200 ppm Ti with respect to the termination temperature of UST above the liquidus temperature. Commercial purity Al inoculated with 50 ppm Ti and 200 ppm in the as-cast condition reduces the grain size from the order of millimetres to ~300–150 μm respectively. Reducing the UST termination temperature close to the liquidus temperature further decreases the grain size at low refiner addition (50 ppm Ti). When the addition level is increased to 200 ppm, better refinement is achieved at higher temperatures. A significant difference in refinement is noted at 10 s UST at 700 °C, where 200 ppm of Ti produces excellent refinement compared to 50 ppm of Ti addition. This difference is reduced when the UST termination temperature reaches the liquidus temperature. For the known distribution of TiB_2_ particles in the AlTiB master alloy and grain size relationship established with a range of wrought Al alloys [65], the dispersion of nucleant particles after the application of UST were characterised using the inter-particle distance ($x_{sd}$). It has been found that the grain size obtained after UST is much smaller than that predicted by $x_{sd}$, suggesting that a larger number of potent substrates are distributed homogeneously and simultaneously activated for better refinement [46,47]. 

Figure 6b shows the refinement of Mg-Zr alloys (plotted for nominal composition). Increasing the addition of Zr from 0.2 to 1.0 wt.% increases the liquidus temperature of the Mg-Zr alloys from 650 °C to 653 °C. UST is applied from 750°C to 660 °C without affecting the onset temperature of α-Mg nucleation. One of the major issues with grain refinement of Mg alloys with Zr addition is the vast difference in the density (~74%) between Mg and Zr particles at alloying temperatures (750 °C) [5,6]. As a result, most of the added Zr particles from the master alloy settle to the bottom of the crucible and approximately 2.33 wt.% of the Mg-Zr master alloy is needed to produce a final alloy composition of 0.7 wt.% Zr [4,6,66]. This severe loss in Zr increases the cost of the alloying process and reduces the grain refinement efficiency in the as-cast condition. Significant reduction in the grain size is obtained only when the addition of Zr is increased above the peritectic composition to 0.8 and 1.0 wt.% in the as-cast condition. After UST, the refinement has been notably improved at lower additions of 0.4 and 0.5 wt.% Zr. With the analysis of composition for dissolved and undissolved particles, it is observed that the UST increases the efficiency of alloying from 30% in the as-cast conditions to 66% by increasing the amount of dissolved Zr solute in the alloy. Reduction in the settling of Zr particles, finer size distribution and its activation by UST maximises the overall efficiency of grain refinement (< 100 μm grain size) for Zr > 0.5 wt.% [6].

### 4.3. Refinement of Primary Intermetallic Phases 

**Figure 7:**
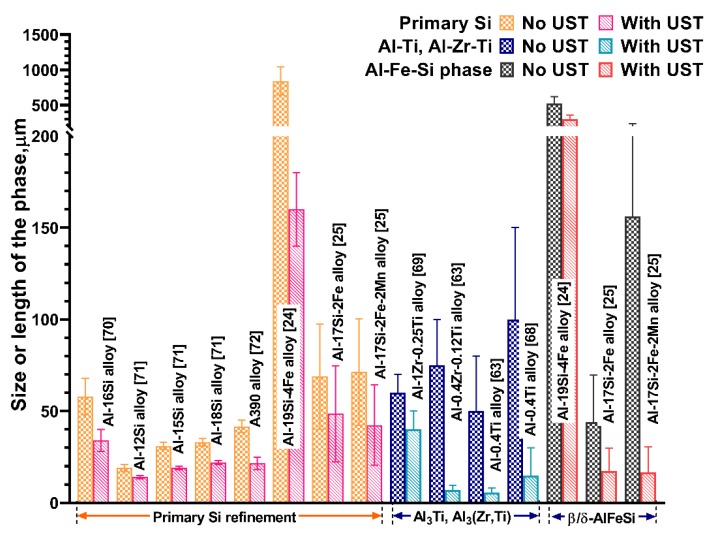
Refinement of primary intermetallic phases after UST reported for selected Al alloys.

One of the major reasons for the increase in the total number density of particles is due to the refinement of primary intermetallic particles by UST and thus increases the population of active substrates for grain refinement [6,47]. It has been reported that the narrow size distribution of refiner particles after UST of Zr added to Mg [6] and Al-Ti-B [67], Al-Zr, Al-Zr-Ti [63] added to Al alloys results in better grain refinement. The refinement of blocky and needle shape primary intermetallic phases such as Al_3_Ti [63,68], Al_3_(Zr, Ti) [63,69], primary Si [70,71,72] and β/δ-Al-Fe-Si [24,25] phases is an added advantage of UST that further improves the mechanical properties of the cast alloys. Figure 7 shows a summary of the common intermetallic phases encountered in commercial Al alloys and their refinement after UST. In Al-Si alloys, both the primary and secondary Si phases are refined. From analysis of cooling curves, it was found that the nucleation temperature of primary Si phase increased [70], indicating that more oxide particles are activated at a smaller undercooling for enhanced nucleation of Si. Direct observation using synchrotron studies reveals that streaming flow, cavitation bubbles and flushing of hot fluid to the roots of the primary phase can cause detachment and remelting for the refinement of primary phases [29,30]. 

Besides refinement in the size of the intermetallic phase, UST also favours the completion of the peritectic reaction reported in Al and Mg alloys [6,24,25]. The sluggish dissolution reaction of Zr in Mg has been improved after UST and increases the solute content to the equilibrium concentration of 0.5 wt.% [6]. Todaro et al. [24] systematically investigated the refinement of primary Si and AlFeSi phases of Al_19_Si_4_Fe alloys with UST. Compared to the as-cast ingot, UST results in the refinement of Fe and primary Si phases, improved their distribution, reduces macro-segregation and large shrinkage cavities. Acoustic streaming produces effective mixing and prevents the settling or floating of intermetallic phases. The peritectic reaction (L + δ-Al_3_FeSi_2_ → β-Al_5_FeSi + Si) in the as-cast condition is incomplete due to the formation of large blocky shape platelets of δ-Al_3_FeSi_2_ and only fine precipitates are expected to undergo complete transformation. After UST, most of the intermetallic particles contain β-Al_5_FeSi and only a few δ-Al_3_FeSi_2_ complex phases exist, indicating that a complete peritectic transformation is favoured. Similarly, it is found that the addition of Mn combined with UST in Al_17_Si_2_Fe(0.5 to 2.0)Mn alloys favoured the transformation to the desirable α-Al_15_(Fe,Mn)_3_Si_2_ phase finely distributed with polygonal morphology [25]. The refinement of primary phase and its morphology is generally reported to be affected by both nucleation and fragmentation effects depending on the temperature range of UST applied during solidification [24,25,68]. 

In alloys containing potent particles (either peritectic systems or when external heterogeneous particles are added), UST can be employed either isothermally above the liquidus temperature or during cooling and terminating UST just above the liquidus temperature. In both these conditions grain refinement is improved by:

(i) De-agglomeration of particles by cavitation and simultaneous improvement in activation (wettability) of the particles by the penetration of parent liquid into the surface defects of the particles [2,47,50,73,74,75];
(ii) Excellent dispersion of the particles by acoustic streaming, as the melt in the liquid state has less resistance to fluid flow [6,47];
(iii) narrow size distribution of nucleant particles throughout the melt and a reduced distance between adjacent potent nucleant particles [6,46,63];
(iv) Reduced loss of inoculants by agglomeration and settling to the bottom of the casting [6];
(v) Fragmentation of large, coarse primary intermetallic phases to fine structures that increase the rate of nucleation of primary grains [24,63,69,70].

## 5. UST Applied from Above to Below Liquidus Temperature during Solidification 

### 5.1. With the Addition Master Alloys Containing Potent Refiner Particles 

**Figure 8:**
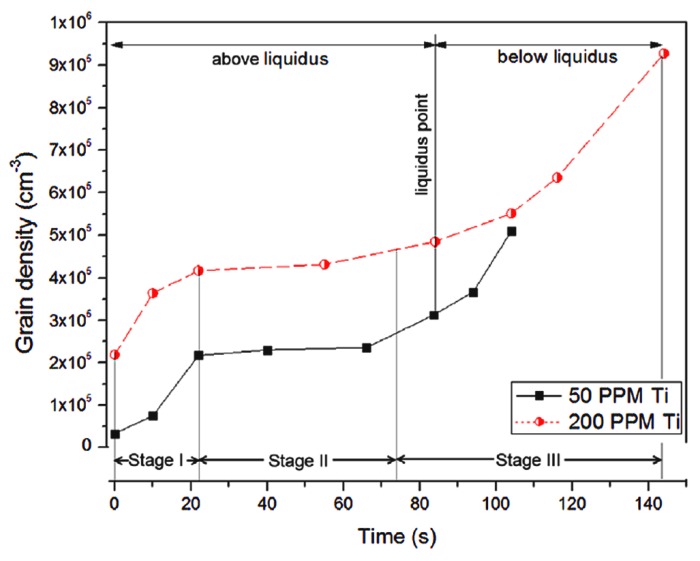
Increase in the grain density with respect to UST time when UST is extended below liquidus temperature of pure Al containing 50 and 200 ppm of Ti [47].

The grain refinement produced by UST when applied in the liquid melt or terminated just above the liquidus temperature mainly depends on the heterogeneous particles. For example, potent particles like TiB_2_ and Zr produce excellent refinement compared to oxide substrates [6,44,46,47]. On the other hand, when the UST temperature range is extended to below the liquidus temperature, the refinement is enhanced to a greater extent even without potent particles or at reduced amounts of inoculant addition. Figure 8 shows that the grain density is remarkably increased when UST is applied below the liquidus temperature in Al containing TiB_2_ particles and the difference in refinement with respect to the amount of Al_3_Ti_1_B master alloy (50, 200 ppm) is almost insignificant. In other words, the amount of grain refinement obtained with 200 ppm could be readily obtained with 50 ppm of Ti when UST continues to be applied below the liquidus temperature for 100 s. It is interesting to note that the grain density increases rapidly until 20 s and reaches a steady state condition until 80 s. This implies that for a given addition amount of refiner, there is a limit to the number density of the particles that can be activated, in which a further activation is possible by extending the UST temperature range to below the liquidus temperature. This is also observed in a Mg-0.2Zr alloy in which a 63% grain size reduction after terminating UST above liquidus temperature has been further increased to 85% when UST is extended below the liquidus temperature for 2 min. This is a significant advantage of UST where the refiner addition can be reduced without compromising the percentage of refinement obtained [6]. 

### 5.2. Pure Metals and Alloys

Without the addition of potent grain refiners, Wang et al. [52] investigated the grain refinement achieved when UST was applied to an Al-2Cu alloy during solidification over different temperature ranges from above the liquidus to complete solidification. It was found that when UST is terminated above liquidus temperature, there is no refinement in the grain size. However, a significant refinement in grain size (150–200 μm) is noted when UST is applied from 40 to 60 °C above the liquidus temperature and continued below the liquidus temperature for 4 min. Reducing the starting temperature of UST to 20 °C above the liquidus or less than that results in the formation of coarse grain structure similar to the as-cast grain structure due to the formation of a solid chill layer beneath the unpreheated sonotrode. With the use of a preheated sonotrode (heated to 285 °C) the formation of a chill layer is avoided, and acoustic streaming was established in the melt to transport grains to form an equiaxed grain structure [38]. 

**Figure 9:**
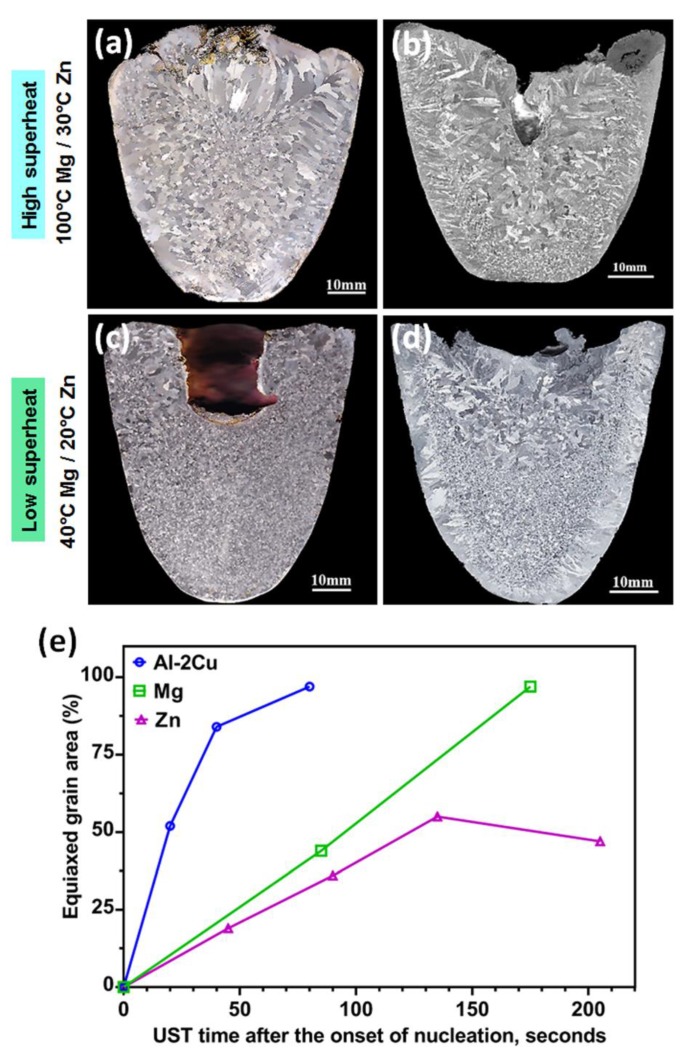
Macrostructures after UST was applied during solidification at (**a**,**b**) high and (**c**,**d**) low superheat (temperature at which UST is turned on above the melting temperature) in (**a**,**c**) pure Mg and (**b**,**d**) pure Zn [43,44]. (**e**) Equiaxed grain area measured from the cross section of the casting with respect to UST time after the onset of solidification [43,44,76].

To understand the mechanism of grain refinement without the interaction of solute or potent particles, pure metals (Mg and Zn) were investigated over different time and temperature ranges. Figure 9 shows the macrostructure refinement after UST was applied to Mg and Zn. Superheat refers to the starting temperature of UST above the melting/liquidus temperature of the alloy. A high superheat temperature (UST turned on at 100 °C above the melting temperature) terminated after 3 min (until complete solidification) produces a coarse and non-uniform grain morphology in Mg (Figure 9a) [44]. When the superheat temperature range is reduced to 40 °C above the melting temperature, the grain refinement is homogeneous and a completely refined structure is obtained throughout the casting with a more uniform distribution of grains (Figure 9c). Interesting insights were revealed from Zn solidification. UST applied from 30 °C above melting temperature for 4 min results in refinement from the as-cast condition, however, the grain structure is completely dendritic in the centre of the casting (Figure 9b). Under similar conditions, a low superheat of 20 °C for 3 min produces an equiaxed zone with non-dendritic grains in nearly half of the casting’s cross section (Figure 9d) [43]. In the as-cast condition, both these metals exhibit a grain size range from 2 to 3 mm on average [43,44,46]. After UST the grain size (in the equiaxed zone) ranges from 160 to 400 μm which is more than 90% reduction in grain size from the respective as-cast conditions. Figure 9e shows a plot of the equiaxed grain area measured from the cross-section area of the casting in pure Mg, Zn and Al-2Cu alloy with respect to UST time [43,44,76]. At a given low-superheat temperature range, increase in the area fraction of equiaxed grains is proportional to the time of UST applied below the liquidus or melting temperature. Depending on the thermal properties of the metals, approximately 1 to 2 min during solidification is enough for Al and Mg to achieve an equiaxed zone throughout the cross section compared to Zn. 

Qian et al. [40] proposed that (in magnesium alloys) these grains are nucleated during UST as a result of cavitation and dispersed into the melt by acoustic streaming. By measuring the grain size with respect to distance from the sonotrode, it is found that the fine grains are observed closer to the sonotrode and the grain size increases with distance from the sonotrode to the bottom of the casting. Therefore, these grains are assumed to nucleate in the cavitation zone beneath the sonotrode and then dispersed into the melt. Due to the attenuation of sound waves, the grain size becomes coarser in the bottom region of the casting near the crucible wall. As grain refinement is observed only when the UST is applied during solidification includes the onset of nucleation, some researchers believe that fragmentation of dendrites by cavitation was the major reason for the grain refinement rather than independent nucleation [2]. The mechanistic viewpoints on grain formation will be detailed in the later sections, however, the grain refinement achieved by extending UST below liquidus temperature is promising because it eliminates or reduces the need for the addition of external particles.

The additional factors (temperature range, time duration and alloying elements) that contribute to deliver grain refinement when UST is applied below the melting or liquidus temperature in alloys are:

(i) Increased number of nucleation events on heterogeneous potent particles compared to UST terminated above liquidus temperature [6,44,47,53];
(ii) Reduction in the temperature gradient of the bulk liquid under the action of acoustic streaming promoting nucleation on potent particles and assists the survival of grains [45,52,57,77];
(iii) Formation of fine non-dendritic grains below the sonotrode at the liquid–sonotrode interface due to the colder vibrating source and then distributed by acoustic streaming into an undercooled melt [6,20,40,43,46];
(iv) Fragmentation of dendrites caused by the interaction of cavitation and acoustic streaming in the mushy zone or at the solid–liquid interface [32,34,35,41,78].

## 6. Grain Formation Mechanisms and Development of the Refined Ingot Structure

### 6.1. Origin of Equiaxed Grains

The dominant mechanism of UST grain refinement is often debated between cavitation causing fragmentation of dendrites [26,27,32,42] and an enhanced nucleation mechanism based on the heterogeneous substrates [1,40,74,79]. Several experiments under a high intensity X-ray synchrotron technique have shown that fragmentation or fracturing of primary phases by cavitation bubbles are responsible for the refinement [34,41,42,78]. However, alloys treated under UST in a crucible exposed to room temperature supports an enhanced nucleation mechanism, where the cavitation induced pressure pulses are expected to increase the rate of nucleation on heterogeneous substrates. The grains produced in this condition show more spherical and non-dendritic morphologies that are much finer than the secondary dendritic arm spacing of the alloys without UST. Therefore, the fragmentation of well-developed grains in this case cannot be the cause of the formation of a fine non-dendritic grain structure. If these grains are expected to be generated by early stage fragmentation then the size of such crystals and its survival rates are questionable [74]. 

**Figure 10:**
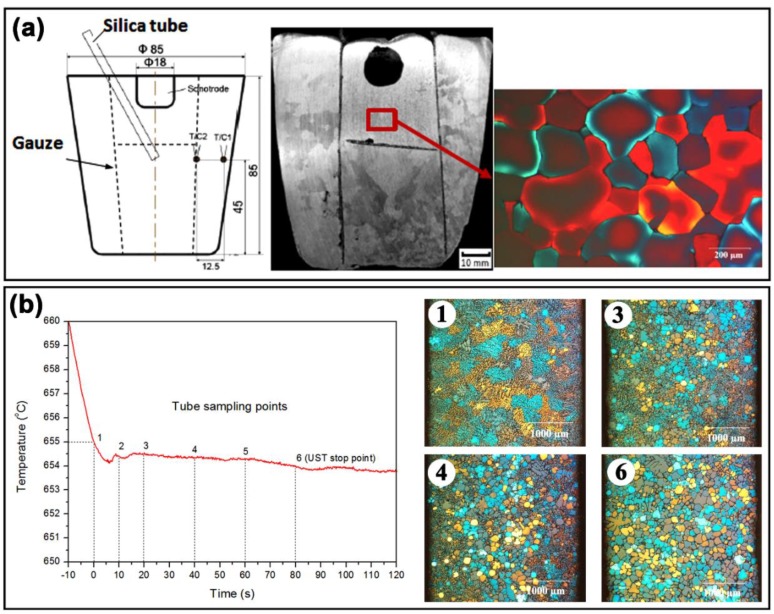
(**a**) Schematic of the UST setup that uses a stainless-steel mesh to capture the grains beneath the molybdenum sonotrode and the resultant macrostructure and microstructure (taken from the gauze area). (**b**) Cooling curve of Al-2Cu alloy with numbers denoting the time at which tube samples were taken during UST and the corresponding microstructures of samples 1, 3, 4 and 6 [76].

To better understand the mechanism of the origin of these non-dendritic grains two approaches were followed to characterize grain formation in the cavitation zone (i) placing a gauze encapsulating the cavitation zone and (ii) using quartz tubes to extract the melt during UST and quenching immediately [76]. Using the gauze setup it was found that the cavitation zone immediately beneath the sonotrode is responsible for grain formation. Figure 10a clearly shows that non-dendritic, fine grains are directly observed at the sonotrode-liquid interface region of the casting. The areas separated by the gauze only show a coarse grain structure (similar to as-cast structure) including adjacent to the mould walls and the top surface of the casting. This confirms that the non-dendritic grains originate in the cavitation zone beneath the sonotrode. 

**Figure 11:**
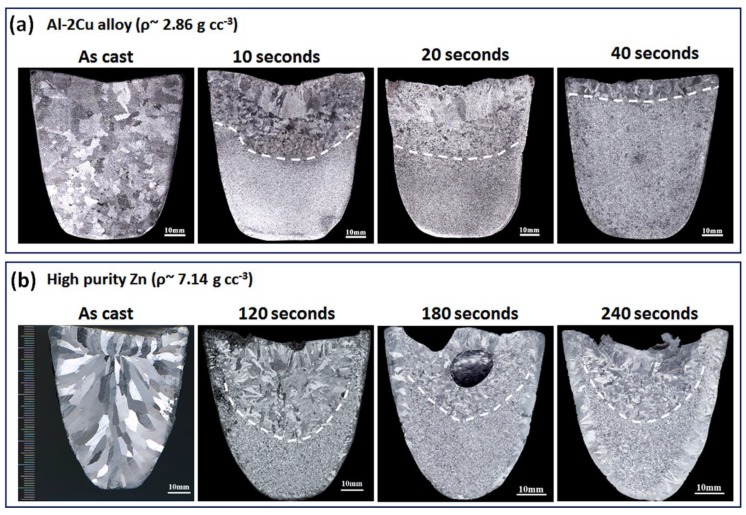
Settling of grains when UST is terminated after various time periods during solidification of (**a**) Al-2Cu alloy and (**b**) high purity Zn [43,76].

Figure 10b shows the points in the cooling curve (1 to 6) where tube samples were taken from the solidifying melt and the microstructures from selected sampling stages (1, 3, 4 and 6). As UST has already started at 40 °C above the liquidus temperature, grains are produced beneath the sonotrode when the temperature reaches the liquidus temperature and then transported into the liquid melt. The microstructure of sample 1 taken at the liquidus temperature shows large dendritic grains formed during quenching of the sample, meaning that the number density of non-dendritic grains is very low. Some non-dendritic grains started to appear in the sample 2 at 10 s after the onset of solidification and continue to increase up to sample 6, at 80 s where the microstructure of the entire tube sample has a large number of non-dendritic grains. Therefore, the grains generated at the start (<20 s) were pushed down by the acoustic streaming force leaving a lower number density of spherical grains at the top of the melt. After 20 s, both the continuous formation of new grains below the sonotrode and the recirculation of the existing grains starts to fill the casting cross section to nearly half of the volume (refer to Figure 11a) and more fine grains start to appear in the top region after 80 s. 

### 6.2. Settling of Grains, Effect of Volume and Height of the Casting

When UST is applied until complete solidification, the whole ingot structure is refined as shown in Figure 9c. As already described in Figure 10b increasing the time gradually increases the number of non-dendritic grains. Figure 11 shows the effect of such grains settling towards the bottom of the crucible when UST is terminated before complete solidification. After the grains are generated in the cavitation zone, the acoustic stream carries these grains to the bottom of the casting [43,76]. Low temperature gradients enhance the survival of a higher number density of grains and it results in the non-dendritic equiaxed zone. Termination of UST at shorter times results in two types of grains above the equiaxed zone (i) rosette or mixed dendritic grains just above the equiaxed layer and (ii) large columnar grains at the top surface of the casting influenced by the radiation heat transfer [76]. Based on the thermal conductivity of the metal, the action of grains filling the macrostructure of the Al-2Cu alloy ingot (Figure 11a) is faster (80 s) than that of pure Zn (240 s, Figure 11b). Furthermore, it is interesting to note that increasing the time from 20 to 80 s (Al-2Cu alloy) produces completely equiaxed grains throughout the cross section of casting whereas in Zn increasing the time duration from 180 s to 240 s results in a similar area of equiaxed zone. Also, the temperature range at which excellent refinement is achieved is 40 °C above the liquidus temperature in Al-2Cu alloy and for Zn it is 20 °C above the melting temperature. This comparison shows that UST produces similar tendencies in grain formation and settling regardless of the type of metal and it only depends on the temperature range of UST. 

**Figure 12:**
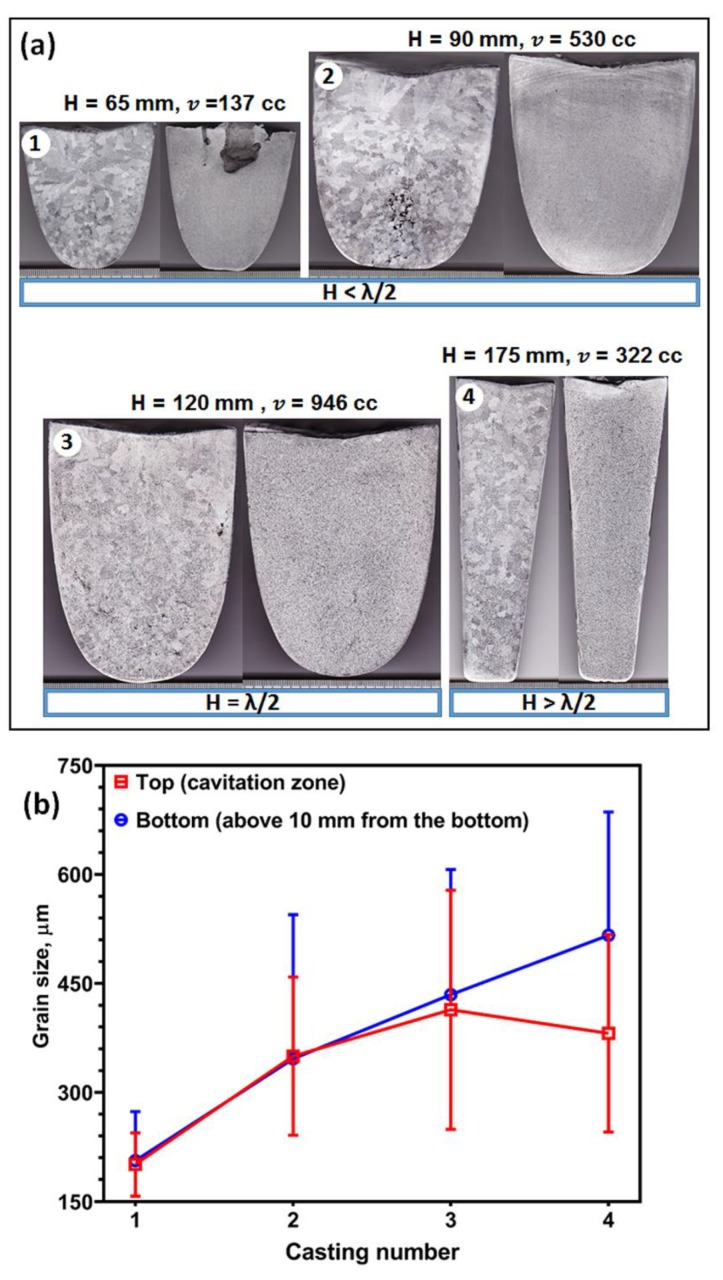
(**a**) Effect of casting volume (v) and height (H) on the macrostructure after UST of an Al-2Cu alloy and (**b**) the grain size measured from the top and bottom of the casting. (λ/2 = 125 mm refers to the half wavelength distance of the sonotorde).

The role of acoustic streaming in transporting the grains for large volumes varying from 137 to 946 cc is shown in Figure 12. The increase in the crucible size led to an increase in the lateral volume in castings 1 to 3 and simultaneously increases the height from 6.5 cm for casting 1 to 12 cm for casting 3. Casting number 4 has the longest distance of 17.5 cm from the sonotrode tip to the bottom of the casting. In all these cases, the sonotrode was immersed into the melt from the top to nearly 1–1.5 cm below the surface. For all the heights of the castings investigated, the application of UST from 40 °C above the liquidus until complete solidification produces refinement throughout the casting’s cross section (castings 1 to 4). However, macro examination of castings 3 and 4 reveals that the grains are slightly coarser than castings 1 and 2. The grain size measured from the top and bottom of castings 1 to 4 is shown in Figure 12b. As the volume and distance increases the grain size steadily increases in the bottom region. For larger castings (2, 3, 4) the grain size in the cavitation zone also shows bigger grains with large deviations compared to casting number 1. In castings 3 and 4, mostly mixed grain structures (grains with rosette and dendritic morphology) are observed throughout the ingot with few non-dendritic grains. As UST is initiated well above the liquidus temperature of the alloy, it is possible that these grains while moving towards the bottom of the crucible, would grow to take a rosette or dendritic form. Increasing the height of the casting above the distance of λ/2 started to show a fading tendency in the degree of grain refinement. Nonetheless, the refinement after UST is significant compared to the respective as-cast conditions.

### 6.3. Evolution of Grain Structure, Morphology of Grains and the Role of Alloying Elements

**Figure 13:**
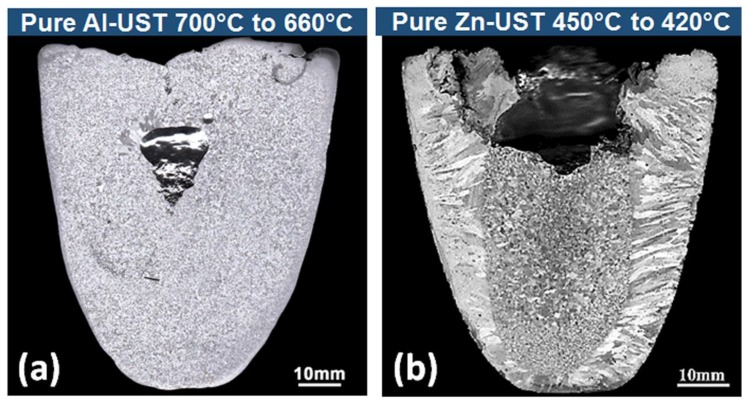
Macrostructures of ultrasonically treated (**a**) pure Al and (**b**) pure Zn.

Depending on the properties of the metal (melting temperature, density and thermal conductivity) significant differences were noted in the UST grain structures of pure Zn, Al and Mg. Figure 13 shows the macrostructure of pure Al and Zn where UST is applied from 40 °C and 30 °C above the melting temperature, respectively, until complete solidification. 

The grain structure in the Al ingot is uniform with only equiaxed grains throughout the cross-section. Pure Mg solidified under similar conditions also shows a fully equiaxed structure for the whole cross section in Figure 9c. On the other hand, both columnar and equiaxed grains were found after Zn solidification under UST. From the macrostructures shown in Figure 9d, the Zn columnar grains grew from ~4.0 mm to 10.6 ± 0.6 mm. According to the grain formation mechanism explained in the Figure 10a these grains filled the small volume of Mg and Al alloys within shorter durations. Due to the larger solidification interval and low melting temperature of pure Zn, any grains created above the melting temperature have a greater chance of remelting. While applying UST below the melting temperature, acoustic streaming reduces the steep temperature gradients in the centre of the melt and the relatively colder zones of the mould started to nucleate columnar grains. These columnar grains have finer width (~0.7 mm) and are numerous along the mould wall compared to the as-cast condition of pure Zn (~3.0 mm) [43]. The increase in the length of the columnar grains growing perpendicular to the direction of the sonotrode indicates that (i) the temperature ahead of the melt is lowered by the acoustic streaming and (ii) there is no obstruction to the continued growth of the columnar grains by the circulating grains. Therefore, fine grains that are formed during this condition are expected be lower in number density and tend to settle quickly towards the bottom of the crucible, allowing the columnar grains to grow from the side wall without impingement.

**Figure 14:**
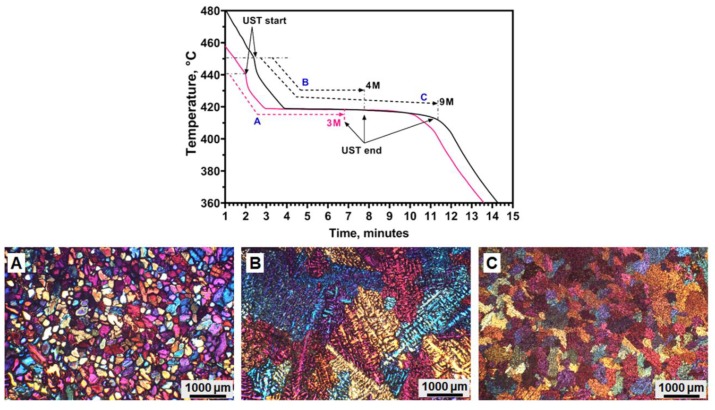
Cooling curve of pure Zn showing three different temperature ranges of UST application **A**, **B**, **C** and their corresponding microstructures taken from the centre of the casting.

Comparison of grain structures with respect to the temperature range of UST for pure Zn is shown in the Figure 14. The cooling curve in Figure 14 shows three ranges of UST A, B and C. An unpreheated titanium sonotrode was used in all these experiments. The microstructures from each casting condition taken from the centre of the casting is shown in Figure 14A–C. 

The low temperature range of UST for a shorter time (440 °C-3 min) results in non-dendritic grains in microstructure A. Increasing the temperature range to 450 °C for a longer time (4 or 9 min) results in either coarse dendrites (B) or equiaxed grains with dendritic morphology (C). It should be noted that the equiaxed grains in A are completely non-dendritic whereas the grains in C are dendritic equiaxed. 3 min of UST produces non-dendritic grains in nearly half of the cross section, because of the low superheat temperature range (A). However, 9 min UST at a slightly higher temperature range produces only dendritic grains (C) in the equiaxed zone and promotes columnar grain growth from the mould wall (Figure 13b). These grain structures show that the formation of non-dendritic grains is related to the low-superheat temperature range of UST. When a higher starting temperature is used, the sonotrode is heated to a higher temperature and results only in dendritic grains. Such clear observations noted in pure Zn are not found in pure Mg or Al and its alloys during UST solidification. 

**Figure 15:**
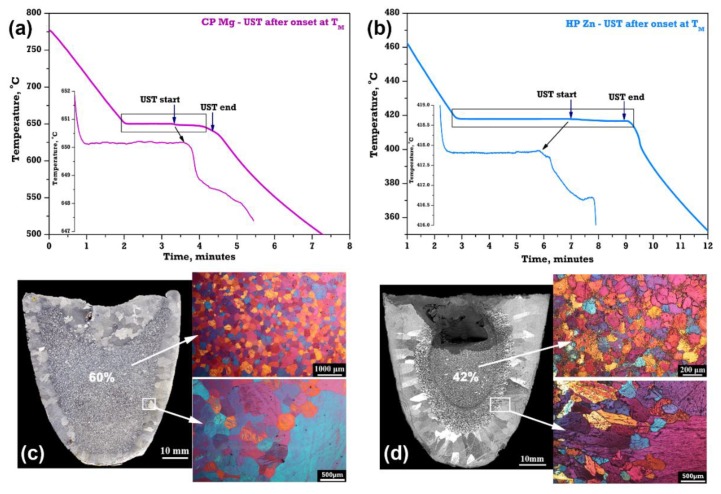
(**a**,**b**) Cooling curves, (**c**,**d**) grain structures in the macrostructure and microstructure of pure (**a**,**c**) Mg and (**b**,**d**) Zn.

To further understand the grain formation effect of an unpreheated sonotrode, UST is applied to pure metals just before the completion of solidification in the equilibrium melt as shown in Figure 15. 

The cooling curves of Mg and Zn in Figure 15a,b show that UST is applied only at the end of complete solidification without affecting the onset of nucleation. Thermocouples were placed slightly offset to the sonotrode (Figure 15a,b) and it was found that there is a significant drop in temperature of the pure metal (~1 to 1.5 °C). As explained in the Figure 10, the grains generated below the sonotrode are pushed downwards into the equilibrium melt to create equiaxed grains in nearly 40% to 60% of the cross section within 1 to 2 min of UST. Microstructures observed in the centre of the casting shows that these grains in both Mg and Zn castings were non-dendritic (Figure 15c,d). The forced downward movement of the fine grains due to acoustic streaming impinge on the columnar grains to form the columnar to equiaxed transition. Therefore, considering the above discussion and the solidification conditions, fragmentation of existing dendrites is less likely to be a significant contributor to the refinement. 

**Figure 16:**
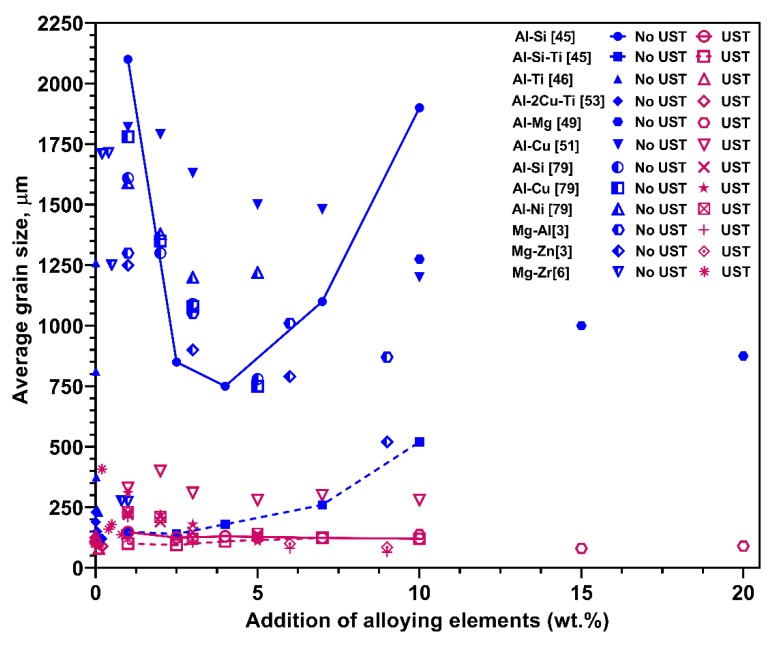
Role of alloying elements on the grain refinement achieved when UST is applied during continuous cooling from above to below the liquidus temperature.

Figure 16 shows the effect of important alloying elements reported in Al and Mg alloys for grain refinement after UST under similar casting conditions. Incremental additions of solute Mg [49], Cu, Ni [79] and Si (< 4 wt.%) [45] to Al alloys produces an average grain size less than 400 μm after UST. When grain refiners are present (TiB_2_ [46,47,53] and Zr particles [1,6]) the effectiveness is further improved even at low additions. It is well-known that the addition of Si > 3–4 wt.% increases the grain size in Al alloys, where the addition of Al-Ti-B refiners cannot produce significant refinement in the as-cast condition [18,19]. The grain coarsening behaviour of Al alloys containing Si without and with Ti is shown by solid lines in Figure 16. UST, on the other hand, refines the grain size in both these alloys (Al-Si and Al-Si-Ti [45]) at the temperature range of 40 °C above the liquidus temperature to complete solidification. Research on Mg alloys (containing Al [80] and Zn [3]) has also shown similar results for UST refinement as a function of solute concentration.

## 7. Interpretation and Application of the Interdependence Model for Solidification under UST

The Interdependence model [15] is a useful framework for analysing the factors that can be controlled in order to optimise an alloy’s grain size $\left(d_{gs}\right)$ by facilitating nucleation. The model is described by Equation (4).

(4) $$d_{gs}=\frac{D\cdot z\Delta T_{n-min}}{vQ}+\frac{4.6D}{v}\cdot (\frac{C_{l}^{*}-C_{0}}{C_{l}^{*}\cdot (1-k)})+x_{Sd}$$

where *D* is the solute diffusion coefficient, $\Delta T_{n-min}$ is the nucleation undercooling required to nucleate on the most potent particle, *z* is the incremental amount of $\Delta T_{n}$ that needs to be generated by constitutional supercooling $\left(\Delta T_{CS}\right)$ for a subsequent nucleation event to occur, v is the growth rate of the grain–liquid interface, $C_{o}$ is the alloy composition, $C_{l}^{*}$ is the composition of the liquid at the interface and k is the partition coefficient. The three terms calculate the elements that make up the grain size such that:

(5) $$d_{gs}=x_{CS}+{x^{\prime }}_{dl}+x_{sd}$$

where $x_{CS}$ is the growth of previous grain to generate $\Delta T_{CS}$ = $\Delta T_{n}$*,* $x{’}_{dl}$ is the length of the diffusion field to where $\Delta T_{n}$ is achieved, and $x_{sd}$ is the average distance to next most potent particle. The first two terms ($x_{CS}$ *+* $x{’}_{dl}$) represent the nucleation free zone $x_{NFZ}$. Note that $x{’}_{dl}$ is controlled by $x_{CS}$ that establishes the value of $C_{l}^{*}$ in Equation (4). In order to reduce the grain size either, or both, of $x_{NFZ}$ and $x_{sd}$ need to be decreased.

The following discusses how these factors are relevant to solidification under UST. From the results described in the previous sections, Q is clearly important and can be readily manipulated. When Q becomes very large $x_{NFZ}$ will tend to zero such that the number of nucleation events corresponds to $x_{sd}$ thus the particle number density controls the amount of nucleation. If the nucleant particles have a very high potency tending towards epitaxial nucleation then $\Delta T_{n}$ tends to zero and thus $x_{NFZ}$ tends to zero. This effect has been demonstrated by the addition of niobium boride particles to an Al-Si alloy where the effect of Si poisoning is eliminated because the niobium boride particles are large and have a very good orientation relationship with aluminium thereby reducing $x_{NFZ}$ to a small value [81]. The interfacial growth rate v will be relatively slow as the cooling rate is low and growth occurs near the liquidus temperature. The solute diffusion rate in the liquid, D, may be enhanced by convection associated with acoustic streaming. However, acoustic streaming has a much more significant effect on the temperature gradient as discussed in Section 3. Thus, the term z in Equation (1) tends to zero because the temperature gradient becomes flatter.

Considering the factors that can be controlled the most important are Q by adding growth restricting elements to the alloy, reducing z through acoustic streaming, and reducing $\Delta T_{n}$ by adding potent nucleant particles. Also, $x_{sd}$ can be reduced by increasing the particle number density of these potent nucleants. 

The results from the UST studies conform to the expectations of the Interdependence model. However, this is surprising since the majority of nucleation events occur in the cavitation zone directly under the sonotrode and not in the bulk of the melt. So why is the Interdependence model effective in predicting significant refinement when UST is applied? The answer lies in consideration of the role of acoustic streaming. Because acoustic streaming flattens the temperature gradient in the bulk of the melt, the melt cools with essentially the same amount of undercooling throughout the casting. Therefore, when the grains formed in the cavitation zone are swept into the melt they move into a melt that is undercooled. Also, for alloys, the solute rejected during grain growth creates a constitutionally supercooled layer which protects the grains from remelting [82]. The higher the value of Q the faster CS is generated providing greater protection from remelting. Therefore, the combination of a low temperature gradient and high Q are critical for the survival of grains leading to a finer grain size. If this situation is satisfied the next biggest effect is the addition of a high grain number density of potent particles to reduce $x_{sd}$.

**Figure 17:**
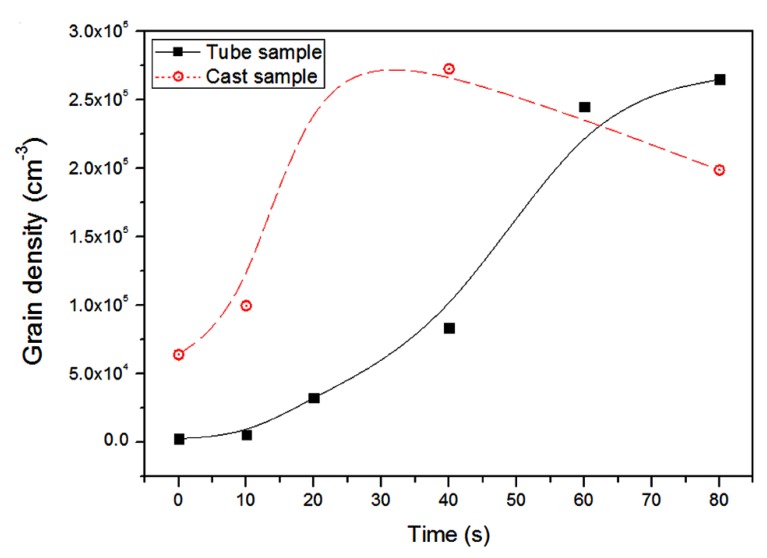
Comparison of grain density measurement of castings (Figure 11a in [76]) and tube samples (Figure 10b in [76]).

$x_{sd}$ has a different meaning for the UST conditions used in our experiments. Because nucleation of equiaxed grains occurs under the sonotrode and not in the bulk of the melt, $x_{sd}$ is defined by the grain number density and not by the number density of potent particles that are able to be activated. Based on the study of grain formation, just below the sonotrode the grain density increases with time during UST as shown in Figure 17. However, in the bulk melt the grain number density initially increases quickly due to fewer small grains with plenty of room to move and grow eventually reaching a maximum after about 30 s when the density becomes higher where grain to grain interactions in the melt become common. After 30 s the number of grains keep increasing while the size of the equiaxed zone increases as shown in Figure 11, but the grain number density does not change significantly. This means that the value of $x_{sd}$ changes during solidification and it is difficult to predict these changes in a casting as $x_{sd}$ also decreases due to the settling of grains towards the bottom of the casting and increases in the top region of the casting due to the depletion of grains. 

**Figure 18:**
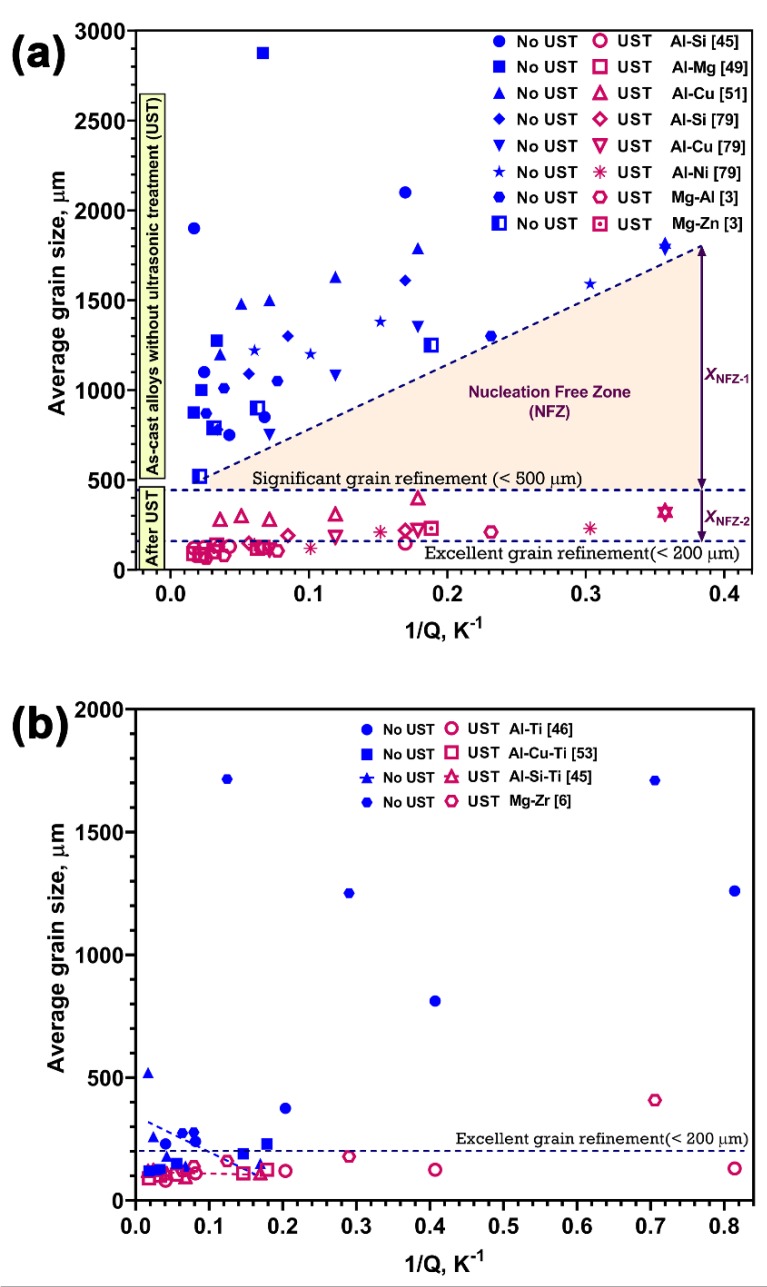
Grain size vs. 1/Q graphs for (**a**) eutectic and (**b**) peritectic alloys solidified under UST.

In plots of grain size versus 1/Q (Figure 18a,b) $x_{sd}$ corresponds to the final grain number density after UST was terminated and settling has finished. Because $x_{sd}$ under UST conditions is based on grains rather than particles the difference with changes in alloy composition are relatively small. Therefore, the role of settling is very important in controlling the grain size as highlighted by Figure 12a. Adapting the Interdependence model to take settling into account is a challenge due to density differences between liquid and grains (e.g., the density differences over a range of Al-Cu compositions) can change dramatically from promoting settling to resulting in floating of grains [83], and between different alloy systems. Despite this difficulty the Interdependence model is still a useful tool for determining the mechanisms controlling grain size.

The following three examples used the Interdependence model to determine the mechanisms responsible for the grain sizes achieved. Figure 18a shows a plot of grain size versus 1/Q for eutectic systems reported for Al and Mg alloys. As these alloys have no active potent substrates for nucleation in the as-cast condition, grain sizes were larger. UST was applied to these alloys from above to below the liquidus temperature during solidification. After UST the grain size was significantly reduced (<500 μm) in low solute containing alloys and for Q values exceeding 10 K the grain refinement becomes excellent (<100 μm). The shaded region between the as-cast and UST refined alloys is $x_{NFZ}$ where $x_{NFZ-1}$ and $x_{NFZ-2}$ highlight the difference between the dilute and high solute alloys, where dilute alloys show grain sizes in the range of 200 to 400 μm. It is interesting to note that grain refinement in conventional casting conditions is largely dependent on Q values with a steeper slope, however, after UST the trend of refinement appears to be much flatter regardless of the type of eutectic forming solutes. Figure 18b shows the effect of Zr solute and particles in Mg and Ti solute and TiB_2_ in Al alloys after UST. Due to the potency of particles and higher Q values, most of the data points in the as-cast condition fall into the significant refinement range (<100 μm), except at very low additions. An increase in the intercept is noted for Al-Si alloys containing Ti due to Si’s poisoning effect, however, UST produces excellent refinement of those alloys.

During UST, the possibility of the formation of new grains is increased rapidly in the cavitation zone [43,44,76] and also by cavitation as a result of physical fragmentation effects [32,33,41,42]. In alloy systems containing potent particles such as Mg-Zr alloys that solidify as equiaxed grains in the as-cast condition, it is assumed that UST preferentially activates nucleation on the Zr particles rather than fragmentation [6,82]. Using the number density of the particles ($N_{v}$) and the weight fraction of particles ($w_{p}$) estimated through chemical analysis, Equation (4) was modified to:

(6) $$d_{gs}=\frac{1000}{\sqrt[{3}]{w_{p}.N_{v}}}+\frac{D}{v}\left[\frac{0.719\cdot z}{d_{p}\cdot Q}+\frac{4.6\;(C_{l}^{*}-C_{ο})}{C_{l}^{*}\cdot (1-k)}\right]$$

where $d_{p}$ is the diameter of the Zr particles. Using Equation (6) the predicted grain size versus 1/Q slope of Mg-Zr alloys after UST is found to be in good agreement with the experimental values 

For the AlTiB master alloy addition to Al alloys, incorporating the effect of Ti solute and TiB_2_ particles, a simplified form of Equation (4) can be expanded as [46]:

(7) $$d_{gs}=\frac{1000}{\sqrt[{3}]{{\left(w_{p}\right)}_{Al3TiB}.N_{vm}}}+5.6\left[\frac{D\cdot z\cdot \Delta T_{n}}{v\cdot Q}\right]=\frac{32}{\sqrt[{3}]{{\left(w_{p}\right)}_{Al3TiB}}}+\frac{652}{Q}$$

**Figure 19:**
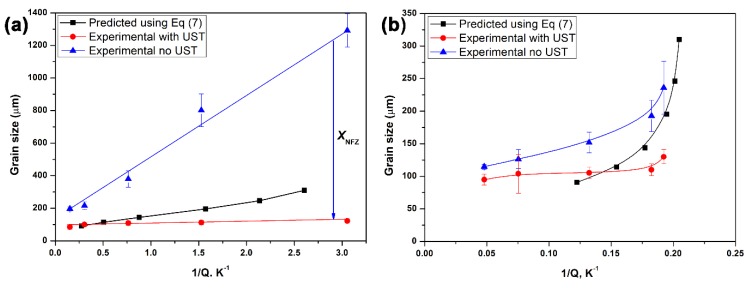
Prediction of grain size using Equation (4) for (**a**) Al-Ti and (**b**) Al-2Cu-Ti alloys with and without UST [46].

Here the weight percentage $w_{p}\;\left(TiB_{2A}/TiB_{2MA}\right)$ is the ratio of the actual amount of TiB_2_ added to the alloy to weight percent of TiB_2_ present in the master alloy and $N_{vm}$ is the number density of TiB_2_ particles in the master alloy. The slope and intercept values in Equation (7) have been previously quantified for a wide range of Al alloys after TiB_2_ addition. Figure 19 shows that the effect of the predicted grain size values with the UST processed Al and Al-2Cu alloys after the addition of Al_3_Ti_1_B master alloy [46].

The grain size values predicted from Equation (7) have low values of the slope because it assumes that all the added particles were active, however, the experimental result for pure Al (Figure 19a) shows a steeper slope (larger grain size) indicating the $x_{NFZ}$ is larger in the as-cast condition. The prediction trend in Figure 19b for Al-2Cu alloy lies close to the experimental curve, which indicates that the presence of solute Cu facilitates the activation of more TiB_2_ particles to reduce the $x_{NFZ}$ in the as-cast condition. The grain refinement observed after UST is much finer than the grain size predicted using Equation (7) indicating that more particles are activated to effectively reduce the $x_{NFZ}$ even at low Q values. An intersection of the predicted curves with UST refinement indicates the maximum refinement condition, above which the refiner and UST produces a similar degree of refinement in Ti added Al alloys. 

In the above two cases (Zr in Mg and TiB_2_ particles in Al alloys) the grain refinement after UST was likely to be affected by the potent particles in the range of 0.2 to 2.5 μm. Research by Dieringa et al. [50] showed that the addition of AlN nano-particles with a size range of 20 to 160 nm (with a mean size of 80 nm) to an AM60 Mg alloy produced excellent refinement with a grain size of 85 μm after UST in the liquid only. Unlike the case of UST during solidification below liquidus temperature where acoustic steaming affects the temperature gradient and the nucleation undercooling of particles, the grain refinement in AM60B-1%AlN composites after UST is terminated and then poured into a mould, is only affected by the distribution of particles and constitutional supercooling. By assuming that the largest particles (in this case it’s 162 nm with a spacing of about 2 μm) are more likely to nucleate grains, and that $x_{NFZ}$ for constant Q is about 85 μm whereas Equation (4) predicts $x_{NFZ}$ would be larger than 600 μm, suggests that $x_{NFZ}$ is affected by D and/or v according to Equation (8).

(8) $$x_{nfz}=\frac{D}{v}\left[\frac{z\cdot \Delta T_{n}}{Q}+\frac{4.6\;(C_{l}^{*}-C_{ο})}{C_{l}^{*}\cdot (1-k)}\right]$$

Since, the parameters inside the brackets in Equation (8) are almost constant, the reduction of $x_{NFZ}$ in this case is expected as a result of change in either D or v. With the measured undercooling of 14 K after addition of the AlN particles [50], a growth rate of approximately seven times faster than the conventional rate would be needed to achieve the grain size of 85 μm. However, no data is available to support this mechanism. As the diffusion field contains rejected solute and a high number density of AlN nano-particles, solute diffusion will be affected by the presence of nano-particles [84,85]. After reducing the value of D from $5\times 10^{-9}\;m^{2}\cdot s$ to $7\times 10^{-10}\;m^{2}\cdot s$ in the Equation (8), $x_{NFZ}$ predicts the measured grain size after UST of this alloy suggesting that the change in diffusion coefficient could dramatically influence the grain structure. 

## 8. Summary and New Insights 

From the analysis of the results of this research and for the casting conditions applied in our experiments, the following provides a generalised description of the key mechanisms affecting the formation of refined grains under UST conditions.

(i) Little refinement is obtained for pure metals and eutectic alloys when UST is applied in the liquid melt. When inoculants or nanoparticles are added to the melt or primary intermetallic and peritectic phases form UST distributes the particles uniformly throughout the melt, breaks up agglomerates of particles and enhances the wetting of particles by the melt. These benefits are realised over a certain UST time for a constant refiner addition, after which no further improvement is obtained. The subsequent solidification occurs as in normal casting processes and the level of refinement achieved can be indicated by the Interdependence model. Application of UST in this temperature range can be readily implemented as part of a foundry’s melt treatment process.
(ii) Nucleation of equiaxed grains occurs directly under the sonotrode when UST is applied below the melting point or liquidus temperature. The strong ultrasonically-induced convection transports these grains into the bulk melt while UST continues to produce new grains. The low temperature gradient generated by the acoustic streaming undercools the bulk of the liquid. Refinement can be obtained for pure metals as well as alloys. Alloys with growth restricting solute generate constitutional supercooling around the growing grains providing good protection from remelting further enhancing the level of refinement. The addition of particle inoculants such as TiB_2_ and Zr, provides an increase in the number of nucleation events particularly in alloys with a low value of *Q*.
(iii) The grains formed under the sonotrode are produced at approximately a constant rate implying that the number of grains in the bulk melt are also increasing at about the same rate. The size of the equiaxed region continues to increase until there are enough grains to fill the casting cavity. This process takes about 80 s for Al alloys and over 150 s for Mg and Zn alloys when cast in the standard size ingots.
(iv) Terminating UST at shorter times shows that the suspended grains sink settling on the bottom of the casting after termination occurs. The settling impedes the growth of adjacent grains and columnar grains growing from the mould wall as they pack together. However, for the same UST conditions, when the ingot height is increased the strength of convection decreases with distance from the sonotrode tip due to attenuation and continued settling, at possibly a slower rate, is due to gravity. Thus, the settling grains have more time to grow so that the final grain size is larger. However, refinement still occurs but not to the same extent as in the smaller castings.
(v) UST diminishes the difference due to variation in alloy composition (i.e., *Q* values) due to a much flatter temperature gradient that decreases the size of the nucleation free zone to a very low value (during traditional casting practices the alloy’s *Q* value is a dominant factor in controlling grain size). This implies that nucleation is now controlled by the grain number density although a small effect of alloy composition remains evident (and in some conditions composition still has a significant effect [1]). These observations indicate that the interpretation of the term *x_Sd_* in the Interdependence model is different for UST conditions. *x_Sd_* is set by the number of grains accumulating in the melt as they are ejected from under the sonotrode whereas for normal casting conditions *x_Sd_* is related to the number density of particle substrates that are able to be activated as nucleants within a bulk melt.

## 9. Directions for Future Research

While the application of UST in the liquid state has already been shown to be industrially useful [34], the application of UST across the liquidus shows potential as a method of obtaining very fine grain sizes but is much more difficult to implement commercially. With this in mind, consideration of the findings and mechanisms described in this paper suggest opportunities for future research. 

Firstly, this work has highlighted that settling and casting size have a significant effect on the degree of grain refinement achieved. Work is needed to quantify the rate of grain formation during UST and the effect of settling on the grain size across the as-cast macrostructure. Related to this is the effect of density differences between grains and liquid on grain size as this will affect the degree of settling or a tendency for grains to float once UST is terminated. Further development of the Interdependence model as applied to UST conditions is needed to improve the prediction of the amount of refinement that can be achieved for particular alloys under specific casting conditions. Computer simulation of micro- and macro-structure development needs to take into account the movement of grains with convection and settling. As it was found that maintaining the sonotrode temperature below the liquidus temperature produced non-dendritic grains while, in the case of Zn, sonotrode heating occurred that reduced the number of non-dendritic grains, it would be worth exploring these effects on larger volumes or over longer times with a water-cooled sonotrode. In parallel, the effect of material properties such as thermal diffusion rate and heat capacity need to be understood to explain the formation of columnar grains enabling tailoring of alloy composition and casting conditions to prevent their formation. Our work has not been focused on the actual mechanisms of nucleation under the sonotrode. Real-time synchrotron studies have focused on cavitation. If this approach could detect nucleation events and the initial growth of grains (e.g., dendritic or non-dendritic) in real time it would clarify which of the proposed mechanisms in the literature are dominant. 

## Figures and Tables

**Table 1:** List of the experiments undertaken at The University of Queensland (UQ) and allied research groups at RMIT University and Helmholtz-Zentrum Geesthacht (HZG).

Alloy Type	Family of Pure Metals and Alloys	Composition (wt.%)	Summary of the Investigation Undertaken with UST	Ref.
Pure metals	Al	-	Evolution of grain structures with respect to temperature range and time duration by keeping the UST power constant	[46]
	Mg			[44]
	Zn			[43]
Eutectic alloys	Al-Cu	2, (1, 2, 3, 5, 7, 10)	Temperature range, time, solute effect and sonotrode preheating	[51,52]
	Al-Mg	5, 10, 15, 20	Role of solute, analysis of mechanism using IDMª	[49]
	Al-Si	1, 2.5, 4, 7, 10	Role of Si and its mechanism	[45]
Peritectic alloys	Al-Ti	0.005, 0.01, 0.02, 0.05, 0.1	Role of Ti, Si, Cu and TiB_2_ particles, temperature range, mechanisms of refinement using IDMª	[46,47]
	Al-Si-Ti	Si (1, 2.5, 4, 7, 10), Ti (0.1)		[45]
	Al-Cu-Ti	Cu (2), Ti (0.005, 0.05, 0.1, 0.2)		[46,53]
	Al-Si-Fe^#^	Si (19), Fe (4)	Primary Si and Al-Fe-Si refinement, peritectic reactions	[24]
	Al-Si-Fe-Mn^#^	Si (17), Fe (2), Mn (0.5, 1, 1.5, 2)		[25]
	Mg-Zr	0.2, 0.4, 0.5, 0.8, 1.0	Settling tendency and size distribution of Zr particles, Zr dissolution and grain refinement mechanism using IDMª	[6]
Nanocomposites	AM60-1% AlN*	Al (6), Mn (0.4)	Grain refinement, mechanical properties, mechanism of refinement using IDMª	[50]

^#^RMIT University, Australia. *HZG, Germany. ªInterdependence Model (IDM).
